# Chronic Pain as a Complication in Open Inguinal Hernia Repair: A Retrospective Study of Consenting Practice in a Single Centre

**DOI:** 10.7759/cureus.23957

**Published:** 2022-04-08

**Authors:** Spencer Probert, Wenyi Cai, Muhammad Rafaih Iqbal, Omotara Kafayat Lesi, Samer-ul Haque, Bryony Lovett, Sarah-Jane Walton

**Affiliations:** 1 General Surgery, Basildon and Thurrock University Hospital, Mid and South Essex NHS Foundation Trust, Basildon, GBR; 2 General and Colorectal Surgery, Basildon and Thurrock University Hospital, Mid and South Essex NHS Foundation Trust, Essex, GBR; 3 General and Colorectal Surgery, Basildon and Thurrock University Hospital, Mid and South Essex NHS Foundation Trust, Basildon, GBR; 4 Colorectal Surgery, Basildon and Thurrock University Hospital, Mid and South Essex NHS Foundation Trust, Essex, GBR

**Keywords:** hernia mesh, health care law, general surgery, chronic pain, complication, consent and capacity in uk law, patient consent, inguinal hernia repair

## Abstract

Introduction

Inguinal hernia repair is one of the most commonly performed procedures in general surgery in the United Kingdom. Chronic pain as a long-term postoperative complication of this procedure has been extensively documented in the literature. However, this complication is often undisclosed during the consenting process. This omission impairs the patients’ informed decision-making process. The Montgomery *v* Lanarkshire Health Board case, in 2015, changed the way in which patient consent is viewed legally. This has made proper consent practices more important to surgeons undertaking procedures.

Aim

The objective is to assess if there has been an improvement in consenting practices by comparing consent forms from 2015 (the year of the Montgomery ruling) and 2019, specifically in regard to the risk of chronic groin pain following open inguinal hernia repair with mesh.

Methods

This was a retrospective review of patients who underwent open inguinal hernia repair using a prosthetic mesh in 2015 and 2019. The medical records were retrieved on the trust’s electronic medical record system using the patient's hospital number. The following parameters were obtained: patient demographics, preoperative clinic letters, operation notes and consent forms. The clinic letters and consent forms were systematically reviewed for any mention of chronic groin pain.

Results

In 2015 and 2019, 163 and 56 open inguinal hernia repairs with mesh were performed, respectively. The median age of patients was 63 (28-88) and 64.5 (19-88) in the respective years. Throughout both years there was a predominance in male patients, and the majority of cases were performed on an elective basis. Consent for chronic pain was present in 60.7% and 62.5% of cases in 2015 and 2019, respectively (p=0.055).

Conclusion

Despite the importance of adequate consenting practice, we found no significant improvement in consenting practice for chronic pain following open inguinal hernia repair in the four years following the Montgomery ruling.

## Introduction

Inguinal hernia repair is a surgical procedure used to repair a fascial or visceral protrusion through a weakness in the lower abdominal wall. It is one of the most commonly performed procedures in general surgery in the United Kingdom (UK), with almost 100,000 cases carried out in the National Health Service (NHS) per annum [1]. This procedure can be done in both open and laparoscopic approaches. In the UK, the repair is most commonly done with mesh augmentation to reinforce the posterior wall of the inguinal canal as it has been shown to reduce the rate of recurrence compared to hernioplasty without mesh [2,3]. The majority of inguinal hernia repairs in the UK happen as elective day cases [4]. Although it is considered to be a straightforward procedure, it is still not without risk. Overall, the procedure itself carries a very low mortality rate (estimated to be 0.004%) in well-resourced settings (i.e. developed countries) [5]. The overall complication risk is approximately 3%, with this estimation rising to 8% with certain patient- and procedural- related factors, such as ASA grades and urgency of the procedure [5]. Early post-operative complications include bleeding, infection, seroma and venous thromboembolism. Recurrence, chronic pain and testicular dysfunction are recognised late complications [6]. Given any of these complications will adversely affect the patient’s quality of life either immediately after the surgery or as an unforeseen event in the future, it is of paramount importance to provide the patient with an adequate amount of information during the consenting process, thus allowing individuals to make informed decisions and exercise their autonomy.

Chronic pain after inguinal repair surgery is defined as groin pain persisting beyond three months [7]. An incidence ranging from 30%-40% has been reported in the literature [6,8,9] and it can be severe in 1%-3% of the patients [10]. Its pathophysiology remains poorly understood and it is difficult to identify individuals who may have a higher risk of developing chronic pain postoperatively [6]. Chronic pain as an entity is included on the list of complications in the guidelines on inguinal hernia management published by the Royal College of Surgeons of England and yet, it is often omitted as a serious complication on the consenting form for inguinal hernia repair [11,12]. A study has found that clinicians, regardless of seniority, achieved high compliance at consenting for immediate complications (e.g., infection and bleeding) but late complications that occur outside the immediate postoperative period tend to remain undisclosed [12].

An omission in risk disclosure impairs the process of informed decision-making and it creates patient dissatisfaction and unfulfilled expectations, leading to litigation in extreme cases. The landmark 2015 Montgomery v Lanarkshire Health Board case, resulted in a change in which patient consent is viewed, legally. Since the 2015 ruling, it was stated that patients should consent to all complications which they may find significant regardless of risk [13]. In the case of chronic pain, a lack of disclosure means that a patient may fail to appreciate the chronic nature of this complication, its treatment options and the potential risks associated with the treatment, such as the burden of polypharmacy [14]. Furthermore, a lack of insight adds to the psychological burden that stems from the unpreparedness for the situation. All of these can mean that the patient’s perceived and actual quality of life is greatly diminished [8]. The consequence of which means that as clinicians, we have failed our duty of care to the patient.

In this study, we aim to retrospectively assess the adequacy of consenting for chronic pain in patients undergoing inguinal hernia repair in both 2015 (the year of the Montgomery ruling) and 2019.

## Materials and methods

This was a retrospective review of the consecutive patients who underwent open inguinal hernia repair using a prosthetic mesh for a 12-month period in both 2015 and 2019. In selecting the years of study, 2015 was selected as this was the year of the Montgomery ruling; 2019 was selected as this allowed enough time to lapse to enable the widespread distribution of the outcome of this ruling, and the adjustments required in consenting practice. A more recent year could not be used due to the impact of COVID-19 on the elective surgery lists in the United Kingdom.

The patients were identified using the operation-specific code on the Operating Room Management Information System (ORMIS) software. Patients were excluded if they were under the age of 18, had an inguinal hernia repair without a mesh, or had a recurrent or bilateral inguinal hernia repair. The medical records were then retrieved on the trust’s electronic medical record system using the patient's hospital number and the following parameters were obtained: patient demographics, preoperative clinic letters, operation notes and consent forms. The clinic letters and consent forms were systematically reviewed for any mention of chronic groin pain (neuropathic pain and neuralgia were also accepted terms).

All results were compiled and analysed in Microsoft Excel. Pearson’s chi-squared test was used to determine the statistical significance of the quantitative data obtained. A statistical significance was set at p < 0.05.

The study was registered locally as an audit and quality improvement project. Data were collected based on theatre records, following this all our data analyses and further work involved no patient identifiable data, and no patient contact was established throughout this study. Hence, IRB was not required.

## Results

**Figure 1:**
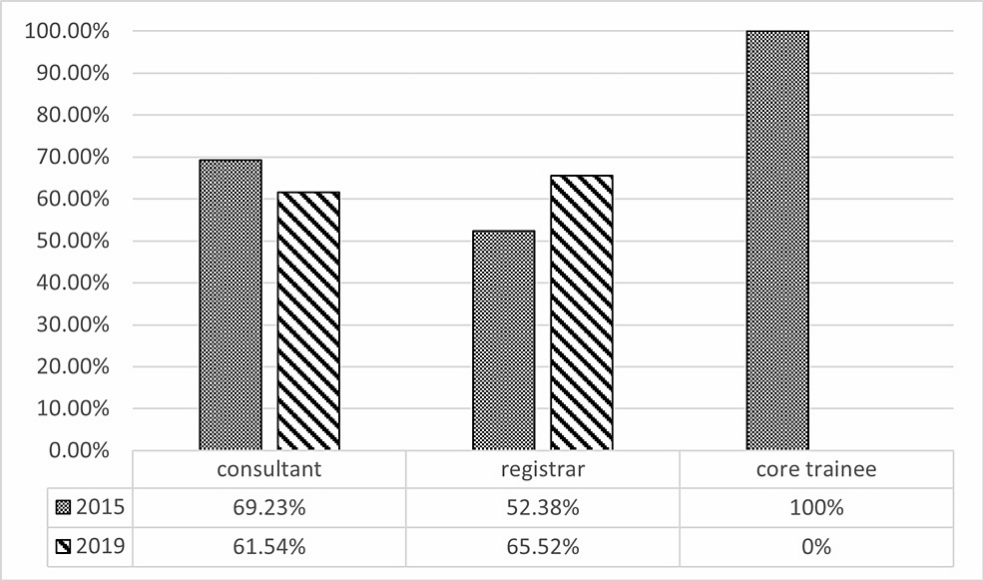
Grade of surgeon consenting for inguinal hernia repair, percentage of consent forms in which chronic groin pain is mentioned. Figures directly compared between 2015 and 2019.

A higher number of open inguinal hernia repairs with prosthetic mesh were performed in 2015 as compared with 2019 (163 v 56). The median age was 63 years (range 28-88 years) and 64.5 years (range 19-88 years) in 2015 and 2019, respectively. In both years, the predominance of cases involved male patients (95.1% and 91.1%). The majority of the cases were elective (94.5% in 2015 and 82.1% in 2019) while emergency cases made up nine and 10 cases each in 2015 and 2019, respectively. The most common American Society of Anaesthesiologists grade (ASA) for patients in both years was 2 (Table 1). The grade of a surgeon in the preoperative clinic letter was comparable between consultants and registrars in both 2015 and 2019. Mention of chronic pain in the preoperative clinic letter was comparable in both years (21.42% and 28.26%), the difference in these two values was found to be statistically insignificant (p=0.334) (Table 2). The grade of surgeon consenting the patient was made up of predominantly registrars in both 2015 and 2019 (51.53% v 51.78%), followed by consultants (47.85% v 46.42%). Patients consented to chronic pain in 60.7% and 62.5% of cases in 2015 and 2019, respectively. The increase of 1.8% is not statistically significant (p=0.055) (Table 3). Figure 1 shows the consenting practice of each grade of the surgeon by looking at the percentage of consent forms where chronic pain is present. Consultants had the most positive outcome in 2015 with 69.23% of patients consented for chronic pain. Registrars followed with 52.38%. In 2019, consultants dropped to 61.54% and registrars increased to 65.52%.

**Table 1:** Patient demographics, the proportion of cases performed electively and American Society of Anaesthetists Grade (ASA).

	2015	2019
Total patients	163	56
Age (median, range)	63 (18-88)	64.5 (19 - 88)
Male (n, %)	155 (95.1)	51 (91.1)
Elective cases (n, %)	154 (94.5)	46 (82.1)
ASA (n, %)		
1	62 (38.03)	16 (28.57)
2	73 (44.78)	27 (48.21)
3	27 (16.56)	12 (7.36)
4	1 (0.61)	1 (1.78)

 

**Table 2:** Mention of chronic pain in preoperative clinic letter with breakdown by grade of assessing surgeon.

	2015	2019	P-value
Grade of surgeon (n, %)			
Consultant	81 (52.59)	21 (45.65)	
Registrar	73 (47.40)	25 (54.34)	
Mention of chronic pain	33 (21.42)	13 (28.26)	0.334

 

**Table 3:** Grade of surgeon completing consent form and mention of chronic pain in the consent form.

	2015	2019	P-value
Grade of surgeon completing consent form (n, %)			
Consultant	78 (47.85)	26 (46.42)	
Registrar	84 (51.53)	29 (51.78)	
Core trainee	1 (0.61)	1 (1.78)	
Mention of chronic pain in consent (n, %)	99 (60.7)	35 (62.5)	0.055

 

## Discussion

Chronic groin pain after open inguinal hernia repair with mesh is a known complication that is infrequently discussed with the patient. It can be debilitating and can significantly affect the quality of life.

In a study of consenting practice at a single trust in 2006, Hoosein et al. found that, in specific regard to chronic pain, consultants consented in 16% of cases and registrars in 13.2% of cases [12]. Our study showed a large improvement in comparison to these values. However, as there is a risk of litigation if this risk is not documented, 100% of patients should consent for chronic pain.

Our results also show that complications, whilst written on the consent form, are not always discussed or documented at the preoperative clinic appointment, as evidenced by clinic letters. These preoperative appointments are crucial in providing patients with all of the information they require in making an informed decision about the treatment pathway ahead. Adequate documentation of this interaction is important in maintaining good record keeping, should any legal action follow.

The 2015 Montgomery v Lanarkshire Health Board case was a turning point in regard to informed consent in the UK. This case involved a pregnant woman of small stature with diabetes who subsequently had a difficult labour due to shoulder dystocia. This led to a hypoxic insult which ultimately resulted in her infant developing cerebral palsy. The lawsuit came about as a result of the patient not being informed about her specific risks, and she stated that if she was aware she would have opted for caesarean delivery. The ruling sided with the patient and stated that clinicians should warn patients not only about the risks they felt were important but also those that would be important to the patient, irrespective of complication rate. It had now become imperative for clinicians to warn patients of material risks, where materiality was defined as - “a reasonable person in the patient’s position would be likely to attach significance to the risk, or the doctor is or should reasonably be aware that the particular patient would be likely to attach significance to it” [13].

With this legal outcome occurring in 2015 we would have expected an improvement in consenting practice in the years proceeding. In regard to open inguinal hernia repair, chronic pain as a long-term complication is a common complication (30%-40%) that can have debilitating effects [6,8,9]. As mentioned, the pathophysiology of developing chronic pain remains poorly understood. This pain may be caused by perioperative damage to the nerves, which may include a meshoma or entrapment within suture material [15]. A 2013 meta-analysis found that there was a reduction in chronic pain in the use of glue over suture fixation [16]. Another study evaluated the impact of mesh weight and found less incidence of chronic pain with lighter mesh material [17]. The difficulty in identifying the exact cause, and which patients will develop chronic pain, creates a challenge in therapeutically managing chronic pain. Due to the prevalence and severity of this complication, it is clear that patients would likely attach significance to it. Therefore, in line with the ruling of the Montgomery case, it would be imperative for clinicians to inform patients about the risk of developing chronic pain following open inguinal hernia repair.

Our study, however, found no significant improvement in consenting practice for chronic groin pain after open inguinal hernia repair with mesh in comparing consent forms from 2015 (the year of the Montgomery ruling) and 2019. Newsome et al. identified a number of barriers to adequate consenting practice. These can be divided into patient-related and clinician-related factors. Patient-related factors include emotional stress, level of education, language barriers, older age and preference for a paternalistic approach. In looking at clinician-related factors, time pressures and lack of awareness can have an impact. Time pressures can be either external or directly related to the physiological status of the patient and possible need for emergency procedures [18]. Poor consent is attributed to about 10% of all the complaints and about 6% of malpractice claims [19]. NHS resolution data shows that “failure to warn” cost the NHS approximately £51.5 million in 2017-2018 [20]. Hence consent process should be improved and patients should be made aware of all the potential risks involved.

## Conclusions

Chronic pain following open inguinal hernia repair is a known and well-documented complication that is historically known to be poorly consented for. With the developments from the Montgomery v Lanarkshire Health Board case, it is more important than ever to consent patients for all potential risks. Despite this, our study has shown no significant improvement in consenting practice in the four years since the Montgomery ruling. A large proportion of patients remain uninformed of the risk of chronic pain following inguinal hernia repair. Given this is a complication patients would attach significance to, we are failing to meet the legal requirements for consent. Further studies could evaluate consenting practices on an international scale and explore further the barriers to adequate consenting practice.
